# Sustainable Formulation of Chewing Candies Using Liver Hydrolysates with Antioxidant and Antimicrobial Properties

**DOI:** 10.3390/microorganisms13081882

**Published:** 2025-08-12

**Authors:** Ignė Juknienė, Naga Pavan Kumar Reddy Jonnagiri, Irena Mačionienė, Gintarė Zakarienė, Jūratė Stankevičienė, Ingrida Sinkevičienė, Vitalijs Radenkovs, Vaida Andrulevičiūtė, Gintarė Zaborskienė

**Affiliations:** 1 Department of Food Safety and Quality, Lithuanian University of Health Sciences, Veterinary Academy, Tilzes St. 18, LT-47181 Kaunas, Lithuania; igne.jukniene@lsmu.lt (I.J.); naga.pavan.kumar.reddy.jonnagiri@stud.lsmu.lt (N.P.K.R.J.); gintare.zakariene@lsmu.lt (G.Z.); jurate.stankevieciene@lsmu.lt (J.S.); 2 Food Institute, Kaunas University of Technology, Radvilėnų pl. 19C-404, LT-50254 Kaunas, Lithuania; irena.macioniene@ktu.lt; 3 Department of Biochemistry, Faculty of Medicine, Lithuanian University of Health Sciences, LT-47181 Kaunas, Lithuania; ingrida.sinkeviciene@lsmu.lt (I.S.); vaida.andruleviciute@lsmu.lt (V.A.); 4 Institute of Horticulture (LatHort), Graudu Str. 1, LV-3701 Dobele, Latvia; vitalijs.radenkovs@lbtu.lv; 5 Research Laboratory of Biotechnology, Division of Smart Technologies, Latvia University of Life Sciences and Technologies, Rigas Str. 22B, LV-3004 Jelgava, Latvia

**Keywords:** functional chewy confection, porcine liver hydrolysates, enzymatic hydrolysis, sustainable food formulation

## Abstract

This study aimed to develop innovative functional gummy candies enriched with protein hydrolysates derived from porcine liver, enhancing their antioxidant and antimicrobial properties. First, the overall consumer acceptability (OA) was assessed to determine the most suitable combination of gummy matrix components. Selected combinations were then analyzed for antioxidant activity (ABTS•+, DPPH•), antimicrobial effects, microbiological safety, and physicochemical characteristics. The incorporation of liver hydrolysates significantly increased antioxidant capacity. The highest activity was observed in sample GC5Pa24Ag, hydrolyzed with papain for 24 h and formulated with agar, showing ABTS•+ and DPPH• scavenging activities of (67.6 ± 0.98 µmol/g) and (49.14 ± 1.00%), respectively (*p* ≤ 0.05). Pepsin hydrolyzed samples (GC2Pe3Gl, GC2Pe6Gl, GC2Pe24Gl) exhibited significantly larger inhibition zones against *Listeria monocytogenes* ATCC 13932, *Escherichia coli* ATCC 25922, and *Salmonella* enterica *subsp*. enterica *serovar Typhimurium* ATCC 14028 compared to the control (*p* < 0.05). Among all, GC5Pa24Ag demonstrated the broadest antimicrobial activity, with a 29.0 ± 0.2 mm inhibition zone against all tested pathogens. These findings suggest that porcine liver hydrolysates can be successfully incorporated into confectionery products to create functional gummies with potential health benefits, offering antioxidant protection and antimicrobial effects in a consumer-friendly form.

## 1. Introduction

In recent years, the food industry has increasingly focused on developing functional and sustainable products to meet consumer demand for healthier and more environmentally friendly choices [1]. Confectionery products, particularly chewy candies, remain popular worldwide; however, they are often criticized for their high sugar content, artificial additives, and low nutritional value [2]. To address these concerns, innovative strategies aligned with the clean label trend are being explored, emphasizing natural ingredients and environmental sustainability [3].

Often considered mere by-products of slaughter, animal liver, lungs, and tongue are a source of the highest-quality protein and biologically active enzymes. Unfortunately, their potential is often untapped, which is why these organs are usually sold unprocessed and at a low price [4,5,6,7]. However, it should be noted that the liver serves as the primary site for the sequestration and metabolism of xenobiotics. Prolonged exposure to heavy metals and antimicrobials may result in their accumulation within this organ [8,9]. Enzymatic hydrolysis enables the degradation of liver proteins into bioactive peptides with demonstrated antioxidant and antimicrobial activities. These hydrolysates not only help reduce waste but also offer dual functionality: improving food preservation through inherent antibacterial effects and promoting health by combating oxidative stress linked to chronic diseases [10].

Due to these functional attributes, health-promoting effects, and shelf-life extension potential, protein hydrolysates have gained significant attention as natural food preservatives. Enzymatic hydrolysis is the most commonly used method of protein degradation to extract biologically active peptides. For the resulting hydrolysates to be suitable for further use, the hydrolysis conditions are of great importance—enzyme type and concentration, reaction time, pH, and the substrate-to-enzyme ratio [11]. Recent in vitro studies have investigated various meat-derived hydrolysates—including liver hydrolysates—for their antioxidant and antimicrobial activities [12,13]. Moreover, protein hydrolysates may serve as valuable nutritional components in specialized adult diets, infant formulas, and as supplemental protein sources for the elderly, individuals with food allergies, or those with congenital metabolic disorders [14,15].

Peptides exhibiting antioxidant properties are proposed to regulate physiological functions and may be utilized in the formulation of nutraceuticals or functional foods; however, it is crucial to acknowledge that these small peptides and free amino acids also affect the flavor and aroma of the final products during development [16,17]. Protein hydrolysates often have a bitter taste, which can negatively affect the taste characteristics of the final food product. This bitterness is usually associated with the presence of low molecular weight peptides; however, meat contains a variety of proteins, and enzymatic hydrolysis enables the production of a broad spectrum of peptides, some of which can effectively function as desirable bitter taste modifiers [18,19,20]. Consequently, it is essential to utilize taste-masking agents, including natural or synthetic compounds that can mitigate or diminish the severity of bitterness in the final product.

Incorporating such ingredients into confectionery may change the sector by combining sustainability with functionality. Conventional candies depend on artificial preservatives such as BHA/BHT, which pose safety concerns to consumers [21]. Replacing these with natural antioxidants and antimicrobials obtained from liver hydrolysates would be an ideal solution to meet clean label demands, as well as increasing the product’s shelf-life [22].

This study aims to create a sustainable formulation of chewing candies enriched with liver protein hydrolysates and to assess their antioxidant and antimicrobial properties, while also tackling potential sensory issues like bitterness through effective flavor-masking techniques.

## 2. Materials and Methods

**Figure 1:**
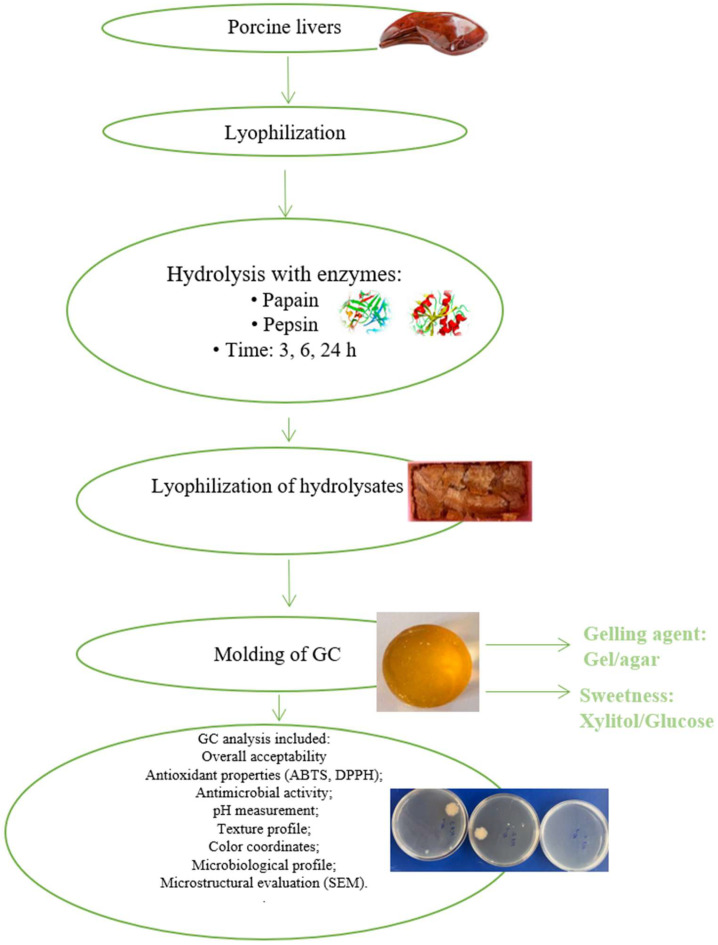
Schematic representation of experimental design.

The main experimental scheme is presented in Figure 1. Porcine liver samples were obtained from pigs aged 6–7 months at two commercial slaughterhouses located in Utena (X) and Alanta (Y), Lithuania. Liver (n = 8) confirmed to be devoid of pathological lesions through post-mortem veterinary inspection were selected for further processing. The samples were transported to the laboratory within 24 h post-slaughter and stored in hermetically sealed containers at a temperature of 4 °C.

The porcine liver was frozen at −35 °C for 3 h using a Liebherr freezer (LGv 5010 MediLine, Baden-Wurtemberg, Germany). Subsequently, it was freeze-dried at −80 °C under a vacuum pressure of 20 Pa using a lyophilizer (Harvest Right, North Salt Lake, UT, USA).

### 2.1. Production of Hydrolysates from Porcine Liver

Freeze-dried porcine liver (n = 8) were ground into fine powder using a laboratory-scale mill (Fritsch Grind Pulverisette 14, Idar-Oberstein, Germany), followed by sieving through a 200 µm mesh. Enzymatic hydrolysis was carried out utilizing papain (activity ≥ 10 units/mg protein) and pepsin (activity ≥ 2500 units/mg protein), both obtained from Sigma-Aldrich (St. Louis, MO, USA). Homogenization of the lyophilized by-products was achieved by mixing with ice in a 1:1 ratio (powder/ice). Optimal enzymatic conditions were applied for each enzyme: papain at 37 °C and pH 6, and pepsin at 37 °C and pH 2.5. The pH was adjusted using 1 N NaOH or HCl as required.

Enzymatic digestion was conducted at three incubation intervals, 3, 6, and 24 h, maintaining a constant enzyme-to-substrate ratio of 10 g/kg (*w*/*w*). Upon completion of hydrolysis, the mixtures were heat-treated at 95 °C to inactivate enzymatic activity, then rapidly cooled in an ice bath. The resulting hydrolysates were filtered using standard filter paper and centrifuged at 4000 rpm for 10 min at 4 °C.

Liver hydrolysates were frozen at −35 °C for three hours using a Liebherr LGv 5010 MediLine freezer (Liebherr, Baden-Württemberg, Germany). Freeze-drying was subsequently conducted using a Harvest Right lyophilizer (84 North St, Salt Lake City, UT, USA) at 80 °C and a vacuum pressure of 20 Pa for a duration of 72 h.

### 2.2. Composition of Raw Materials for Gummy Candy Preparation

**Figure 2:**
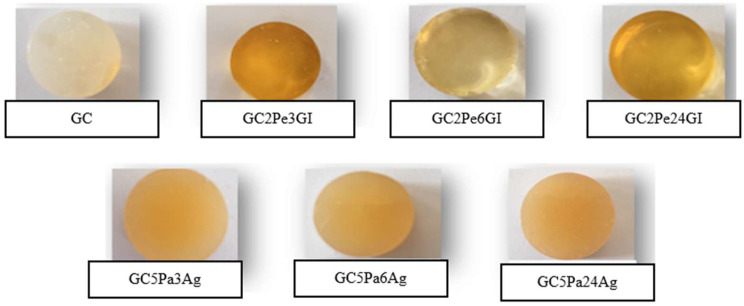
Visual appearance of gummy candies formulated with different protein hydrolysates.

During the research, seven different gummy candy formulations were developed (Table 1). Agar powder (from Gelidium sesquipedale algae; Ag) and gelatin (Gl) were purchased from Rotmanka (Gdańsk, Poland) and Klingiai (Kaunas, Lithuania), respectively. Sweetness was achieved using either xylitol (Natur Hurtig, Nuremberg, Germany) or sugar (Sanitex, Kaunas, Lithuania). Citric acid was obtained from a local retailer (UAB “Maxima LT”, Kaunas, Lithuania) and ascorbic acid was obtained from Valentis (Vilnius, Lithuania). Chewy candies were prepared by incorporating various hydrolysates (2 g or 5 g). To mask undesirable taste notes of hydrolysates, the flavoring agent 23490 Lime flavor (Stockmeier Food GmbH, Zeppelinstraße 7, Herford, Germany) in liquid form was incorporated in water. Initially, the Ag powder was solubilized by heating at 100 °C for 3 min in water. When preparing GC with gelatin, the GI powder was first dissolved in water for 30 min at 30 ± 2 °C, then melted at 80 ± 2 °C. Then, sugar and/or xylitol were added while boiling, and the mixture was further heated with constant stirring. Later, citric acid (or ascorbic acid), hydrolysates, and a flavoring agent were added to the gummy candy mass after thorough mixing; the prepared mixture was poured into silicone molds (circular shape) (ø 1.9 cm, height 1.5 cm); the weight of one gum was 2.05 ± 0.18 g before drying and 2.01 ± 0.12 g after drying (Figure 2).

### 2.3. Analysis of Color Characteristics, Texture, pH, and Overall Acceptability of Gummy Candies with Hydrolysates

The color properties were assessed with a portable colorimeter PCE-CSM5 (PCE Instruments, United Kingdom) featuring CIE L*a*b*C*H* color space choices. In this system, L* denotes lightness, a* denotes the green–red color axis, and b* denotes the blue–yellow color axis. The C* (chroma) value reflects the color saturation, and H* (hue angle) defines the dominant spectral component (red, green, or blue), with values ranging from 0° to 360°.

The texture (hardness) was assessed via a Brookfield CT-3 Texture Analyser (Middleboro, MA, USA).

The pH of the samples was determined according to EN ISO 2917:2002 [23]. A pH meter (Inolab 3, Hanna Instruments, Milan, Italy) was used for the measurements. Before the analysis, the instrument was calibrated using standard buffer solutions with pH values of 4.01 and 7.00 (Sigma–Aldrich, St. Louis, MO, USA). After calibration, the pH electrode was inserted directly into the hydrolyzed chewy candy samples.

The overall acceptability of the prepared gummies with pig liver hydrolysates was assessed by eight trained panel members using the ISO 8586-1 sensory evaluation methodology [24].

### 2.4. Assessment of Free Radical Scavenging Activity Using DPPH• Assay

A standard solution of DPPH radical was prepared by accurately weighing 0.0024 g of DPPH (±0.0001 g), which was dissolved in 80% (*v*/*v*) ethanol using an ultrasonic bath. The solution was then diluted to a final volume of 100 mL in a volumetric flask. The absorbance of the resulting solution was measured at a wavelength of 515 nm, with 80% ethanol serving as the reference solution. Gummy candy extract samples were prepared by dissolving 1 g of the product in 100 mL of distilled water with constant stirring at room temperature for 1 h. The mixture was then centrifuged at 8000 rpm for 10 min at 4 °C. The supernatant was collected, filtered through a 0.45 μm PTFE membrane filter, and stored at −80 °C until further analysis.

The antioxidant activity was determined using the 2,2-diphenyl-1-picrylhydrazyl (DPPH•) radical scavenging assay, with three replicates performed for each extract. The antiradical activity was evaluated based on the proportion of stable DPPH• radicals neutralized by the antioxidant compounds present in the sample. For each measurement, 60 μL of the extract was mixed with 3 mL of a 6 × 10^−5^ M ethanolic DPPH• solution in a 1 cm path-length cuvette. The decrease in absorbance was monitored spectrophotometrically at 515 nm until equilibrium was reached (approximately 30 min). Antioxidant activity was expressed as the percentage of DPPH• radicals inactivated.

$$DPPH•=\left(\frac{Ab-Aa}{Ab}\right)\times 100\%$$

*Ab*—absorbance of the blank sample (t = 0 min); *Aa*—absorbance of the sample with the tested chewing candy extract (t = 30 min)

### 2.5. Assessment of Free Radical Scavenging Activity Using ATBS•+ Assay

Free radical scavenging capacity was evaluated using a spectrophotometric method. A 2 mM stock ABTS•+ solution was prepared and allowed to stand in a dark glass bottle for 16 h. The working ABTS•+ solution was obtained by diluting the stock solution with distilled water to achieve an absorbance of 0.800 at 734 nm. Distilled water was used as the blank (reference solution). Gummy candy extract samples were prepared by dissolving 1 g of the product in 100 mL of distilled water with constant stirring at room temperature for 1 h. The mixture was then centrifuged at 8000 rpm for 10 min at 4 °C. The supernatant was collected, filtered through a 0.45 μm PTFE membrane filter, and stored at −80 °C until further analysis.

The antioxidant activity of biologically active compounds was assessed using Trolox as the standard antioxidant. For the assay, 3 mL of the working ABTS•+ solution was mixed with 30 µL of the test extract. The mixture was kept at room temperature in the dark for 1 h. Each gummy candy extract was tested in triplicate. The decrease in absorbance at 734 nm was measured using a spectrophotometer.

$$\mathrm{TE}\;(\mathrm{ABTS}•+)=\frac{C\times V}{m};\;\mathrm{µmol}/g$$

*C*—Trolox concentration (µmol/L), determined from the calibration curve; *V*—extract volume (L); *m*—accurately weighed amount of raw material (g)

### 2.6. Determination of Antimicrobial Activity of Gelatin-Based Gummy Agar

The antimicrobial properties of gelatin-based gummies were evaluated using the agar well diffusion method. To assess antimicrobial activity, reference strains of conditionally pathogenic bacteria commonly found in food products were used: *Escherichia coli* ATCC 25922, *Listeria monocytogenes* ATCC 13932, *Bacillus cereus* ATCC 11778, *Staphylococcus aureus* subsp. *aureus* ATCC 25923, and *Salmonella enterica* subsp. *enterica serovar Typhimurium *ATCC 14028. The bacterial cultures were pre-cultivated on agar slants in test tubes at 30 °C (for *B. cereus*) and 37 °C (for other strains) for 18–24 h. Then, fresh cultures were used to prepare bacterial suspensions adjusted to 0.5 McFarland standard (approximately 1.5 × 10^8^ CFU/mL). One milliliter of each suspension was pipetted into 100 mL of Mueller Hinton agar (Biolife, Italy), melted and cooled to 45 °C, mixed gently, and poured into Petri dishes (12 mL per dish). After solidification, 8 mm diameter wells were made in the agar. The tested samples were preheated to 37–40 °C, and 50 µL of each sample was dropped into the wells.

The Petri dishes were incubated at the appropriate temperatures, 30 °C for *B. cereus* and 37 °C for the other bacterial strains, for 18–24 h. After incubation, the diameters of the inhibition zones (clear rings around the wells) were measured in millimeters. The positive control was a gummy with citric acid and gelatin, while the negative control was sterile water with gelatin. The experiments were performed in triplicate.

### 2.7. Determination of Antimicrobial Activity of Agar-Based Gummies

The antimicrobial properties of agar-based gummies were evaluated using the following reference bacterial strains: *Escherichia coli* ATCC 25922, *Listeria monocytogenes* ATCC 13932, *Bacillus cereus* ATCC 11778, *Staphylococcus aureus* subsp. *aureus *ATCC 25923, and *Salmonella enterica* subsp. *enterica serovar Typhimurium *ATCC 14028. Petri dishes for the experiment were prepared using the agar well diffusion method. After the agar solidified, the test gummies (dimensions 10 × 10 mm) were placed on the surface of the agar. The Petri dishes were incubated at the appropriate temperatures, 30 °C for B. cereus and 37 °C for the other bacterial strains, for 18–24 h. After incubation, the diameters of the inhibition zones (clear halos around the gummies) were measured in millimeters. The positive control was gummy candies with citric acid and agar, while the negative control was sterile water with agar. The experiments were performed in triplicate.

### 2.8. Microstructural Evaluation of GC with Hydrolysates Using SEM

Cross-sectional examination of the gummy candy samples was performed using a “Mira3” scanning electron microscope (SEM) from Tescan Orsay Holding, a.s. (Brno-Kohoutovice, Czech Republic). The samples were manually cut into small cross-sectional pieces (0.4 × 0.4 cm), mounted onto SEM pin stubs using double-sided adhesive carbon film, and coated with a 6 nm thick gold–palladium layer using a Leica EM ACE600 vacuum sputter coater (Leica Microsystems, Wien, Austria). Both backscattered electron (BSE) and secondary electron (SE) detectors were used. Imaging was performed at 1.0 kx magnification and 5 kV accelerating voltage to allow for accurate characterization of the dimensions and features.

### 2.9. Microbiological Profile

The microbiological quality of gummy candy samples was evaluated by determining the total aerobic mesophilic bacteria count, enterobacteria count, and the number of yeasts and molds. Microbiological testing was conducted on three different storage days: the 1st, 7th, and 21st days. The gelatin- and agar-based gummy candies were stored in hermetically sealed polyethylene bags. Storage temperature was maintained at 18–23 °C, with relative humidity at 60–65%. The samples were protected from direct sunlight.

Sample preparation and decimal dilutions were performed using sterile buffered peptone water following ISO 6887-1:2017 [25] and ISO 6887-4:2017 [26], which provides general and product-specific procedures for preparing confectionery-type samples. From each dilution, 1 mL was plated in duplicate for microbial enumeration. Total aerobic mesophilic bacteria were enumerated using ISO 4833-1:2013 [27] on Plate Count Agar (PCA; Merck, Germany) incubated at 30 ± 1 °C for 72 ± 3 h. Enterobacteria were enumerated according to ISO 21528-2:2017 [28] on Violet Red Bile Glucose Agar (VRBG; Biolife, Italy), incubated at 37 ± 1 °C for 24 ± 2 h. Yeasts and molds were determined using ISO 21527-1:2008 [29] on Dichloran Rose Bengal Chloramphenicol Agar (DRBC; Liofilchem, Roseto degli Abruzzi, Italy), incubated at 25 ± 1 °C for 5 days. The results were expressed as colony-forming units per gram of sample (CFU/g).

## 3. Statistical Analysis

A one-way ANOVA was conducted using SPSS Statistics version 30 to assess whether there were statistically significant differences in the inhibition zones between the tested samples for each bacterial strain. The assumptions of normality and homogeneity of variance were checked before analysis. Tukey’s HSD post hoc test was applied to identify significant pairwise differences between the means of the samples.

## 4. Results and Discussion

### 4.1. Antioxidant Activity (ABTS•+, µmol/g, and DPPH•,%) of Gummy Candies That Contain Liver Hydrolysates and Various Gelling Agents

Table 2 presents the evaluation of the antioxidant properties (ABTS• + and DPPH• free radical scavenging activity) and pH values of gummies enriched with porcine liver hydrolysates and various gel-forming agents. The control gummy candies without hydrolysates (GC) had a lower pH of 3.00 ± 0.02 and exhibited less antioxidant activity in both the ABTS• + (8.52 ± 0.58 µmol/g) and DPPH• (8.62 ± 0.16%) methods. The incorporation of papain hydrolyzed porcine liver into the gummy candy matrix resulted in an increased pH and enhanced antioxidant activity. The most pronounced effect was observed after the longest hydrolysis period (24 h), with the GC 5 Pa 24 Ag sample showing a pH of 3.65 ± 0.03, as well as ABTS• + and DPPH• radical scavenging activities of 67.6 ± 0.98 and 49.14 ± 1.1.0 (*p* ≤ 0. 05). Samples containing pepsin hydrolyzed liver proteins with the gel-forming agent gelatin exhibited significantly lower ABTS• + radical scavenging activity, ranging from 8.91% to 18.31%. These results indicate that liver hydrolysis using pepsin in combination with gelatin as the gel-forming agent was less effective at enhancing the antioxidant properties of the gummies compared to formulations containing papain-derived hydrolysates.

Different enzymes are highly specific for the cleavage of peptide bonds [30]. Therefore, the type of protease is a crucial factor that directly affects the size, amount, composition, and amino acid sequence of the resulting peptides, which in turn influences the antioxidant properties of the hydrolysate [31]. Duan et al. confirmed the trend of our study: liver hydrolyzed with papain showed the best results in DPPH• (19.9–43.4%), depending on the hydrolysis conditions, compared to hydrolysates obtained using pepsin and trypsin [32].

Li et al. conducted studies on porcine collagen hydrolysates and found that antioxidant properties improved with increasing reaction time. The study also utilized various enzymes and a mixture of enzymes that demonstrated the best antioxidant properties [33]. Hidalgo et al. also found in their study that DPPH• radical scavenging activity increased with prolonged hydrolysis time [34]. Verma et al. also observed in their study that the best antioxidant properties were determined after the longest hydrolysis period (6 h) when liver was used as a substrate [35]. Studies have also shown that liver hydrolysates possess higher antioxidant activity than hydrolysates obtained from other tissues, such as the pancreas or colon [36].

### 4.2. Antimicrobial Activity of Gummy Candies That Contain Liver Hydrolysates and Various Gelling Agents

Table 3 presents the antimicrobial activity of gummy candies enriched with porcine liver hydrolysates obtained using the pepsin enzyme and different hydrolysis times. Samples GC2Pe3Gl, GC2Pe6Gl, and GC2Pe24Gl were compared to a control sample (GC), made from a gummy candy containing gelatin and citric acid. The antimicrobial effects of the samples were tested against five foodborne pathogenic bacteria: *E. coli*, *S. aureus* subsp. *aureus*, *L. monocytogenes*, *S. enterica* subsp. *enterica serovar Typhimurium*, and *B. cereus*.

The data indicated that sample GC2Pe6Gl exhibited the strongest overall antimicrobial activity. It demonstrated significantly larger inhibition zones compared to the control sample, GC, against all tested bacteria except *B. cereus*, for which both samples showed equal inhibition (10.0 ± 0.0 mm). The inhibition zones for *E. coli*, *L. monocytogenes*, and *S. enterica *subsp.* enterica serovar Typhimurium* ranged from 15.5 ± 0.3 to 16.0 ± 0.0 mm for GC2Pe6Gl, indicating broad-spectrum effectiveness. The inhibition zone against *S. aureus* subsp*. aureus* was 13.0 ± 0.1 mm, which also exceeded the control.

Sample GC2Pe24Gl showed the most pronounced inhibition against *S. aureus* subsp. *aureus* (17.5 ± 0.3 mm), and strong activity against *S. enterica* subsp.* enterica* serovar Typhimurium (15.5 ± 0.2 mm) but had no observable effect on *B. cereus*.

GC2Pe3Gl demonstrated high activity against *S. enterica* subsp. *enterica serovar Typhimurium* (16.5 ± 0.3 mm) and *L. monocytogenes* (15.5 ± 0.3 mm), as well as a notably increased inhibition zone against *E. coli* (14.5 ± 0.3 mm). However, it showed no effect on *B. cereus* and exhibited the same level of activity as the control against *S. aureus* subsp. *aureus* (10.0 ± 0 mm).

Table 4 presents the antimicrobial activity of gummy candies enriched with porcine liver hydrolysates obtained using the papain enzyme and different hydrolysis times. The only difference between the experimental and control samples was the addition of porcine liver protein hydrolysates. The control sample (GC), which lacked hydrolysates, exhibited the weakest inhibition across all tested pathogens, with inhibition zones ranging from 11.0 ± 0.1 mm (*L. monocytogenes*) to 16.0 ± 0.1 mm (*S. enterica* subsp. *enterica serovar Typhimurium*). The sample GC5Pa3Ag, which contained hydrolysates after 3 h of hydrolysis, demonstrated improved inhibition against *L. monocytogenes* (21.0 ± 0.1 mm), while the activity against other bacteria remained similar to the control, indicating a limited but specific antimicrobial enhancement. Markedly greater antimicrobial effects were observed in samples with longer hydrolysis times. GC5Pa6Ag (6 h) exhibited broad-spectrum activity, with inhibition zones of 29.0 ± 0.2 mm against *E. coli*, 27.0 ± 0.1 mm against *S. aureus *subsp. *aureus*, and 30.0 ± 0.2 mm against *L. monocytogenes*. Similarly, GC5Pa24Ag (24 h) showed the most pronounced activity against *L. monocytogenes* (30.5 ± 0.2 mm) and *S. enterica* subsp. *enterica serovar Typhimurium* (22.0 ± 0.2 mm), as well as substantial inhibition against *E. coli* (29.0 ± 0.2 mm) and *S. aureus* subsp. *aureus* (28.0 ± 0.1 mm). The results suggest that longer hydrolysis significantly enhances the antimicrobial potential of the hydrolysates, particularly against both Gram-negative and Gram-positive bacteria.

The choice of different enzymes and the duration of hydrolysis play a crucial role in the production of antibacterial peptides [37]. These antimicrobial peptides can selectively damage cell membranes, leading to increased membrane permeability, depolarization, dissipation of electrochemical gradients, and ultimately, cell death [38].

Antimicrobial activity depends on the molecular weight of the peptides formed. According to Kim and Wijesekara [39], small-mass peptides (<10 kDa), positively charged and composed of amphipathic molecules—containing both hydrophilic and hydrophobic regions—exhibit pronounced antimicrobial activity [40]. Furthermore, their biological function, including the ability to penetrate microbial cell membranes, depends on the amino acid sequence, lipid membrane composition, and peptide concentration in the environment [41]. A longer hydrolysis time results in greater antimicrobial activity, as it leads to the production of lower molecular weight peptides with enhanced activity against microbial membranes. This is also supported by the study of Borrajo et al., in which the highest antimicrobial effect was observed after 10 h of hydrolysis [42].

Verma et al. [35] found that porcine liver protein hydrolysates obtained using papain exhibited antibacterial activity against *Staphylococcus aureus* and *Bacillus cereus* after 6 h of hydrolysis, whereas no antibacterial effect was observed at the initial time point (0 h) for any of the tested bacteria. This antibacterial effect is believed to be associated with the cationic properties and hydrophobicity of the peptides. Cationic, hydrophobic peptides can effectively interact with the anionic surface and cytoplasmic membranes of microbial cells, causing increased permeability and leakage of intracellular contents, ultimately leading to cell lysis. Additionally, lower molecular weight peptides often interact with membranes more rapidly than peptides of higher mass or longer chain length [4,5,6]. Borrajo et al. reported similar results, where the highest antimicrobial activity was achieved after the longest hydrolysis time of 10 h [42].

A similar trend was observed in our formulated gummy matrices—the longer the hydrolysis time, the more pronounced the inhibition of microbial growth. This highlights the fundamental importance of enzymatic hydrolysis for the efficacy of bioactive compounds.

### 4.3. Texture, Color Parameters, Overall Acceptability, and Microstructure of Gummy Candies Containing Liver Hydrolysates and Different Gelling Agents

Samples marked with different letters in Table 5 indicate statistically significant differences among gummy candy formulations (*p* < 0.05). The color and texture characteristics of hydrolysate-incorporated agar gummies exhibited distinct trends, both aligning with and diverging from the existing literature on confectionery and protein–sugar systems. A general darkening effect, indicated by reduced L* values in hydrolysate-containing samples (excluding GC2Pe6Gl), aligns with previous observations of protein-rich gel systems. Kumkong et al. [43], for instance, reported similar L* reductions in gelatin gummies fortified with whey protein concentrate, attributing this change to the formation of melanoidins via Maillard reactions during storage.

A notable chromatic trend was the concentration-dependent shift in a* values: high-hydrolysate samples (5 g) demonstrated a blue-green tint (lower a*), whereas low-hydrolysate samples (2 g) shifted toward yellow-red tones (higher a* and b*), reflecting differential chromophore development with increasing hydrolysate content. Particularly, samples with low hydrolysate but high glucose content (e.g., GC2Pe3Gl, c* = 18.02) exhibited remarkable color purity. This is consistent with Affes et al. [44], who showed that glucose can enhance color vibrancy by accelerating Maillard reactions in hydrocolloid matrices, even at lower protein concentrations. Their findings emphasize glucose’s role in the generation of Maillard reaction products that intensify both color saturation and clarity.

Moreover, the consistent decrease in hue angle across hydrolysate samples—most pronounced in GC2Pe24Gl (h = 75.77°)—indicates a shift toward yellow hues. This mirrors the results of Wongwiwat and Wattanachant [45], who observed similar hue reductions in protein-rich chicken meat jerky as a result of Maillard browning. These parallel findings reinforce the central role of Maillard-driven pathways in color development within protein–sugar hydrocolloid matrices. Texture profiles showed contrasting effects based on hydrolysate concentration and type. The highest hardness in GC5Pa24Ag (4.63 mJ) suggests that papain hydrolysates at high doses reinforce agar networks, possibly via hydrophobic peptide–agar interactions.

This observation aligns with studies on papain-treated soy protein isolate, where enzymatic hydrolysis enhanced gel characteristics by promoting stronger protein network formation [46]. Conversely, the marked softening in 2 g hydrolysate samples (e.g., GC2Pe3Gl, 0.56 mJ) contrasts with studies where hydrolysates increased gel strength [47]. This discrepancy may result from competitive water binding: low hydrolysate doses in gelatin-containing systems may disrupt agar helix aggregation, as gelatin’s random coil structures reduce network density. The control’s intermediate hardness (3.33 mJ) highlights the concentration-dependent dual role of hydrolysates, both structuring and destabilizing. The results show that hydrolysates in gummies interact more complexly than just adding up, depending on the type of hydrolysis, the dose, and the reducing sugar content.

The highest overall acceptability score (8.5 ± 0.5) was recorded for sample GC2Pe3Gl, which contained pepsin hydrolyzed pig liver and gelatin as a gel-forming agent, and the hydrolysate content was 3 g. Samples GC2Pe6Gl and GC2Pe24Gl also received favorable scores and were rated better than the control. Also, these samples showed significantly higher acceptability (*p* < 0.05) compared to all three papain hydrolyzed samples—GC5Pa3Ag, GC5Pa6Ag, and GC5Pa24Ag—which had an increased hydrolysate content (5 g). These results suggest that a lower amount of hydrolysate combined with gelatin is the most acceptable to consumers. Protein hydrolysates are often characterized by a bitter taste, which presents a challenge for product formulation. Therefore, effective masking strategies and careful optimization of the hydrolysate concentration are essential to ensure acceptable sensory properties in the final product. The taste of hydrolysates can be masked using gelatin, modified starch, and malic acid or citric acid [48,49]. Bertelsen et al. observed that xylitol, sucrose, α-cyclodextrin, and maltodextrin act as taste-masking agents to reduce the bitterness of soy protein hydrolysates [50].

Also, gummy candy samples prepared with agar and higher concentrations of protein hydrolysates exhibited increased textural firmness, which may have contributed to the overall acceptability assessment. Texture is a crucial factor in determining food acceptability, as the textural properties experienced in the mouth significantly influence the pleasure of eating [51,52].

**Figure 3:**
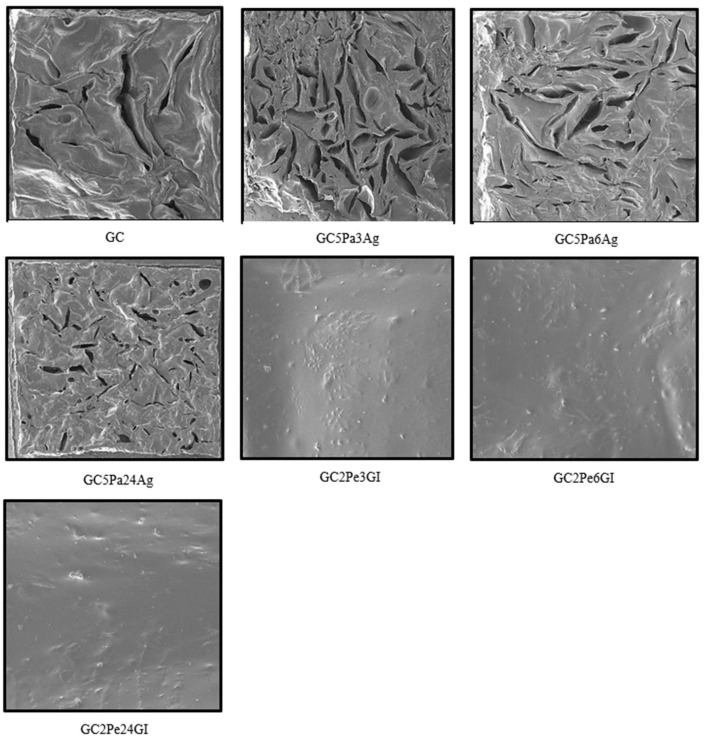
Scanning electron microscope (SEM) images of samples (operating voltage: 5.0 kV; magnification: 1.00 kx; scale bar = 50 µm).

Figure 3 shows the differences in the microstructure of different samples. SEM images show differences in the surface morphology of different GC-based composites. The control sample (GC) has a completely homogeneous, slightly wavy surface without a pronounced pore structure. After adding liver hydrolysates and using agar as a gelling agent in samples GC5Pa3Ag, GC5Pa6Ag, GC5Pa24Ag, the highest microporosity and higher phase separation are observed. Meanwhile, samples with gelatin and liver hydrolysates (GC2Pe3Gl, GC2Pe6Gl, GC2Pe24Gl) are characterized by a homogeneous surface and reduced roughness and porosity, which indicates stronger intermolecular interactions. Such structural differences indicate that the added hydrolysates have a decisive influence on the formation of microstructures in gel compositions.

Rabhani et al. conducted studies and observed differences between agar and gelatin matrices: agar gels are characterized by larger (~370–700 nm) and irregularly rounded pores, while gelatin gels are characterized by smaller (~320–650 nm) and more uniform pores and a more homogeneous microscopic appearance [53]. Maaloum et al. observed that the structure of gels is primarily dependent on the concentration, with the structure becoming more homogeneous as the concentration increases [54]. The arrangement of proteins in gel matrices also depends on the concentration and pore size distribution of the gels [53].

The microbiological quality of the gummy candy samples produced with gelatin or agar was evaluated over a 21-day storage period. Detailed microbial counts for all samples and time points are presented in Table 6. No aerobic mesophilic bacterial growth was detected in any sample on the first day of storage. After 7 days, low bacterial counts (up to 1.70 log_10_ CFU/g) were only observed in the gelatin-based gummies, while the agar-based samples remained free of contamination. On day 21, the gelatin-based samples showed increased bacterial counts ranging from log_10_ 2.07 to 2.86 CFU/g (equivalent to 1.2 × 10^2^ to 7.2 × 10^2^ CFU/g). The agar-based samples continued to exhibit no detectable bacterial growth throughout the entire storage period.

Enterobacteria were not detected in any sample at any storage time point. Yeasts and molds remained below the quantification limit (<1.0 log_10_ CFU/g) in all samples at all time points, indicating good microbiological stability.

Mesophilic aerobic microorganisms, including aerobic bacteria, yeasts, and other microscopic fungi, are a primary cause of microbiological contamination in food products. Their presence is usually linked to microbiologically contaminated raw materials, insufficiently effective processing, or inadequate storage conditions, which together create environments favorable for microbial growth [55]. The size of the total microbial population is also strongly affected by pH: in products with pH < 4.5, the growth and survival of most pathogenic microorganisms are restricted. Under such acidic conditions, acid-tolerant microbes such as yeasts and molds and a few acid-resistant bacterial species predominate [49]. This trend was confirmed in our study as the agar gummies prepared with papain liver hydrolysate had an acidic pH (3.65–3.70), which likely contributed to their superior microbiological profile. Water activity (aw) and moisture content are key factors governing confectionery shelf-life [54]. Kaewpetch et al. showed that gelatin-based gummies possess relatively high moisture, elevating the risk of microbial growth [56]. Vojvodić et al. likewise observed that the high moisture content of gelatin sweets compromises microbiological stability; lowering the moisture level would extend the shelf-life but at the cost of a less desirable texture [57].

**Figure 4:**
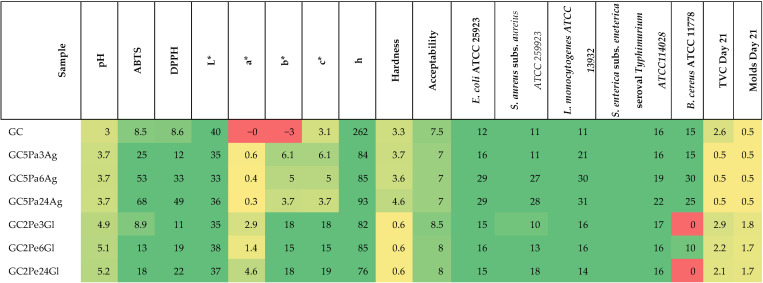
Heat map visualization of key quality parameters of gummy candies enriched with porcine liver hydrolysates. GC—gummy candy; 2.0, 5.0—content of hydrolysates (g); 3, 6, and 24 h—hydrolysis time; Pa—papain hydrolysis; Pe—pepsin hydrolysis; Ag—agar; Gl—gelatin.

The heat map visualization in Figure 4 illustrates the primary quality parameters of gummy candies that have been supplemented with porcine liver hydrolysates. Enrichment with porcine liver hydrolysates enhanced the antioxidant properties of the gummy candies and frequently increased their acceptability, as indicated by the heat map. The samples GC5Pa6Ag and GC2Pe6Gl exhibited the most favorable overall results, as they exhibited a balanced combination of favorable chemical, textural, and sensorial evaluation outcomes.

## 5. Conclusions

The gummies developed with liver hydrolysate could be a healthier alternative to traditional gummies or jelly beans, and their production could enable the sustainable use of by-products. Porcine liver is a significant and promising source of bioactive peptides. The hydrolysis time and enzyme type significantly influenced the properties of gummy candies (*p* < 0.05).

The highest antimicrobial and antioxidant activities were demonstrated by the GC5Pa24Ag sample, which was hydrolyzed for the longest duration (24 h) using the papain enzyme. This sample exhibited the largest inhibition zone diameter (29.0 ± 0.2 mm), indicating its superior antimicrobial efficacy compared to the other tested samples. The choice of gelling agent and sweeteners significantly influenced brightness (L*), yellowness (b*), texture, and overall acceptability. It was observed that no bacterial growth occurred throughout the 21-day storage period when agar was used as the gelling agent.

The practical application of the tested hydrolysates could target individuals with diabetes or those at increased risk, especially type 2 diabetes, which is often linked to obesity and cardiovascular diseases. For this group, a product was developed without simple carbohydrates, using a sweetener and papain hydrolysates with high antioxidant activity. The gel structure was created with plant-based agar. The second gummy formulation could be designed to meet the nutritional needs of rapidly growing children, adolescents, and elderly individuals. It contained easily digestible pepsin hydrolysates, glucose, and animal-derived gelatin—components important for connective tissue regeneration and for supporting growth and recovery processes in the body.

## Figures and Tables

**Table 1:** Composition of gummy candy formulations containing porcine liver hydrolysates.

Gummies Formula	GI, g	Ag, g	Water, mL	Citric Acid, g	Ascorbic Acid, g	Xylitol, g	Glucose, g	Extract Lime, mL	Hydrolysate, g
GC		10	100	1	1	10		0.1	
GC5Pa3Ag		10	100	1		10		0.1	5
GC5Pa6Ag		10	100	1		10		0.1	5
GC5Pa24Ag		10	100	1		10		0.1	5
GC2Pe3GI	10		100		1		10	0.1	2
GC2Pe6GI	10		100		1		10	0.1	2
GC2Pe24GI	10		100		1		10	0.1	2

GC—gummy candies; 2.0, 5.0—content of hydrolysates (g); 3, 6, and 24 h—hydrolysis time; Pa—papain hydrolysis; Pe—pepsin hydrolysis; Ag—agar; GI—gelatin.

**Table 2:** Antioxidant activity (ABTS•+, µmol/g; DPPH•, %) and pH of gummy candies formulated with porcine liver hydrolysates obtained using different enzymes and various gel-forming agents.

Material	pH	ATBS•+ Scavenging Activity	DPPH• Scavenging Activity
GC	3.00 ± 0.02 ^a^	8.52 ± 0.58 ^a^	8.62 ± 0.16 ^a^
GC5Pa3Ag	3.66 ± 0.02 ^a^	24.6 ± 0.93 ^b^	11.93 ± 0.68 ^b^
GC5Pa6Ag	3.70 ± 0.03 ^a^	52.67 ± 0.51 ^c^	33.29 ± 1.07 ^c^
GC5Pa24Ag	3.65 ± 0.03 ^a^	67.6 ± 0.98 ^c^	49.14 ± 1.0 ^c^
GC2Pe3Gl	4.90 ± 0.04 ^b^	8.91 ± 0.25 ^a^	11.26 ± 0.21 ^b^
GC2Pe6Gl	5.05 ± 0.03 ^b^	12.72 ± 0.31 ^b^	19.22 ± 0.11 ^b^
GC2Pe24Gl	5.19 ± 0.03 ^b^	18.31 ± 0.59 ^b^	22.0 ± 1.17 ^b^

^a–c^—mean values in columns with different letters differ significantly (*p* ≤ 0.05). Data are presented as mean (n = 3) ± standard deviation (SD). GC—gummy candies; 2.0, 5.0—content of hydrolysates (g); 3, 6, and 24 h—hydrolysis time; Pa—papain hydrolysis; Pe—pepsin hydrolysis; Ag—agar; Gl—gelatin.

**Table 3:** Antimicrobial activity of gummy candies formulated with gelatin base.

Material	Diameter of Inhibition Zones, mm				
	*E. coli* ATCC 25922	*S. aureus* subsp. *aureus* ATCC 25923	*L. monocytogenes* ATCC 13932	*S. enterica* subsp. *enterica serovar Typhimurium* ATCC 14028	*B.cereus* ATCC 11778
GC	11.0 ± 0.1 ^a^	10.0 ± 0.1 ^a^	11.0 ± 0.1 ^a^	11.0 ± 0.1 ^a^	10.0 ± 0 ^b^
GC2Pe3Gl	14.5 ± 0.3 ^b^	10.0 ± 0 ^a^	15.5 ± 0.3 ^b^	16.5 ± 0.3 ^b^	nd
GC2Pe6Gl	15.5 ± 0.3 ^b^	13.0 ± 0.1 ^b^	15.6 ± 0.2 ^b^	16.0 ± 0.0 ^b^	10.0 ± 0 ^b^
GC2Pe24Gl	14.5 ± 0.3 ^b^	17.5 ± 0.3 ^c^	13.5 ± 0.2 ^c^	15.5 ± 0.3 ^b^	nd

^a–c^—mean values in columns with different letters differ statistically significantly (*p* ≤ 0.05); data are presented as mean (n = 3) ± standard deviation (SD); nd—not detected. GC—gummy candy; 2.0, 5.0—content of hydrolysates (g); 3, 6, and 24 h—hydrolysis time; Pa—papain hydrolysis; Pe—pepsin hydrolysis; Ag—agar; Gl—gelatin.

**Table 4:** Antimicrobial activity of chewing candies formulated with agar base.

Material	Diameter of Inhibition Zones, mm				
	*E. coli* ATCC 25922	*S. aureus* subsp. *aureus* ATCC 25923	*L. monocytogenes* ATCC 13932	*S. enterica* subsp. *enterica serovar Typhimurium* ATCC 14028	*B. cereus* ATCC 11778
GC	12.0 ± 0.1 ^a^	11.0 ± 0.1 ^a^	11.0 ± 0.1 ^a^	16.0 ± 0.1 ^a^	15.0 ± 0 ^a^
GC5Pa3Ag	16.0 ± 0.1 ^b^	11.0 ± 0 ^a^	21.0 ± 0.1 ^b^	16.0 ± 0.1 ^a^	15.0 ± 0.1 ^a^
GC5Pa6Ag	29.0 ± 0.2 ^c^	27.0 ± 0.1 ^b^	30.0 ± 0.2 ^b^	19.0 ± 0.1 ^b^	30.0 ± 0.3 ^b^
GC5Pa24Ag	29.0 ± 0.2 ^c^	28.0 ± 0.1 ^b^	30.5 ± 0.2 ^b^	22.0 ± 0.2 ^b^	25.0 ± 0.2 ^b^

^a–c^—mean values in columns with different letters differ significantly (*p* ≤ 0.05); data are presented as mean (n = 3) ± standard deviation (SD); nd—not detected. GC—gummy candy; 2.0, 5.0—content of hydrolysates (g); 3, 6, and 24 h—hydrolysis time; Pa—papain hydrolysis; Pe—pepsin hydrolysis; Ag—agar; Gl—gelatin.

**Table 5:** CIELab color coordinates of chewing candies formulated with agar base during storage.

Samples	Color Coordinates, NBS					Texture Hardness	Overall Acceptability
	L*	a*	b*	c*	h	mJ	
GC	39.83 ± 0.72 ^a^	−0.02 ± 0.54^c^	−3.03 ± 0.47 ^c^	3.06 ± 0.49 ^c^	262.23 ± 1.74 ^a^	3.33 ±0.06 ^b^	7.5 ± 0.5 ^a^
GC5Pa3Ag	35.26 ± 0.42 ^b^	0.63 ± 0.06 ^b^	6.09 ± 0.04 ^b^	6.11 ± 0.06 ^b^	84.11 ± 0.62 ^b^	3.73 ±0.15 ^b^	7.0 ± 0.8 ^a^
GC5Pa6Ag	32.77 ± 0.76 ^b^	0.43 ± 0.1 ^b^	5.01 ± 0.05 ^b^	5.03 ± 0.04 ^b^	85.33 ± 0.86 ^b^	3.63 ±0.06 ^b^	7.0 ± 0.3 ^a^
GC5Pa24Ag	36.01 ± 1.49 ^b^	0.30 ± 0.02 ^b^	3.74 ± 0.12 ^b^	3.73 ± 0.14 ^b^	92.76 ± 0.57 ^b^	4.63 ±0.15 ^a^	7.0 ± 0.5 ^a^
GC2Pe3Gl	35.06 ± 2.73 ^b^	2.94 ± 0.06 ^a^	17.79 ± 0.15 ^a^	18.02 ± 0.33 ^a^	82.24 ± 2.69 ^b^	0.56 ±0.06 ^c^	8.5 ± 0.5 ^b^
GC2Pe6Gl	38.34 ± 0.58 ^a^	1.35 ± 0.05 ^b^	15.16 ± 0.48 ^a^	15.22 ± 0.48 ^a^	84.93 ± 0.26 ^b^	0.66 ±0.06 ^c^	8.0 ± 0.3 ^b^
GC2Pe24Gl	36.9 ± 0.23 ^b^	4.62 ± 0.33 ^a^	18.08 ± 0.16 ^a^	18.59 ± 0.08 ^a^	75.77 ± 0.96 ^c^	0.60 ±0.10 ^c^	8.0± 0.5 ^b^

^a–c^—mean values in columns with different letters differ statistically (*p* ≤ 0.05); data are presented as mean (n = 3) ± standard deviation (SD). GC—gummy candy; 2.0, 5.0—content of hydrolysates (g); 3, 6, and 24 h—hydrolysis time; Pa—papain hydrolysis; Pe—pepsin hydrolysis; Ag—agar; Gl—gelatin.

**Table 6:** Microbiological quality of gummy candy samples containing liver hydrolysates and different gel-forming agents over 21-day storage.

Total Viable Count (log_10_ CFU/g)			
Samples	Day 1	Day 7	Day 21
GC	<1.0	<1.0	2.55
GC5Pa3Ag	<1.0	<1.0	<1.0
GC5Pa6Ag	<1.0	<1.0	<1.0
GC5Pa24Ag	<1.0	<1.0	<1.0
GC2Pe3Gl	<1.0	1.70	2.86
GC2Pe6Gl	<1.0	<1.0	2.19
GC2Pe24Gl	<1.0	<1.0	2.07
**Molds and Yeasts (log_10_ CFU/g)**			
GC	<1.0	<1.0	<1.0
GC5Pa3Ag	<1.0	<1.0	<1.0
GC5Pa6Ag	<1.0	<1.0	<1.0
GC5Pa24Ag	<1.0	<1.0	<1.0
GC2Pe3Gl	<1.0	<1.0	1.80
GC2Pe6Gl	<1.0	<1.0	1.66
GC2Pe24Gl	<1.0	<1.0	1.74

GC—gummy candy; 2.0, 5.0—content of hydrolysates (g); 3, 6, and 24 h—hydrolysis time; Pa—papain hydrolysis; Pe—pepsin hydrolysis; Ag—agar; GI—gelatin.

## Data Availability

The raw data supporting the conclusions of this article will be made available by the authors on request.
